# A phenomenological approach shows a high coherence of warming patterns in dimictic aquatic systems across latitude

**DOI:** 10.1007/s00227-012-1934-5

**Published:** 2012-05-04

**Authors:** Annekatrin Wagner, Stephan Hülsmann, Lothar Paul, Rüdiger J. Paul, Thomas Petzoldt, René Sachse, Thomas Schiller, Bettina Zeis, Jürgen Benndorf, Thomas U. Berendonk

**Affiliations:** 1 Institute of Hydrobiology, Technische Universität Dresden, 01062 Dresden, Germany; 2 Neunzehnhain Ecological Station, TU Dresden, 09514 Lengefeld, Germany; 3 Institute of Zoophysiology, Westfälische Wilhelms-Universität Münster, 48143 Münster, Germany

## Abstract

To predict the coherence in local responses to large-scale climatic forcing among aquatic systems, we developed a generalized approach to compare long-term data of dimictic water bodies based on phenomenologically defined hydrographic events. These climate-sensitive phases (inverse stratification, spring overturn, early thermal stratification, summer stagnation) were classified in a dual code (cold/warm) based on threshold temperatures. Accounting for a latitudinal gradient in seasonal timing of phases derived from gradients in cumulative irradiation (2.2 days per degree latitude), we found a high spatial and temporal coherence in warm–cold patterns for six lakes (84 %) and the Baltic Sea (78 %), even when using the same thresholds for all sites. Similarity to CW-codes for the North Sea still was up to 72 %. The approach allows prediction of phase-specific warming trends and resulting instantaneous or time-delayed ecological responses. Exemplarily, we show that warming during early thermal stratification controls food-web-mediated effects on key species during summer.

## Introduction

Climatic and large-scale oceanic fluctuations are recognized as important regulatory factors influencing the thermal and structural properties of terrestrial and aquatic ecosystems (Straile 2000; Walther et al. 2002; Sharples et al. 2006; Blenckner et al. 2007). Within the past decades, substantial progress has been made in identifying the effects of climatic forcing on aquatic ecosystems (Straile 2000; Edwards and Richardson 2004; Adrian et al. 2006; Weyhenmeyer 2008; Thackeray et al. 2010; Tian et al. 2011); however, there are limitations in our ability to predict the coherence in local responses to large-scale climatic forcing among aquatic systems across latitudinal gradients (Magnuson et al. 1990; Livingstone and Padisak 2007; Livingstone et al. 2010). From recent studies, it emerged that the degree of coherence among lakes is greatest for conventional climatic variables (such as air temperature) and large-scale driving forces (such as North Atlantic Oscillation (NAO), Pacific Decadal Oscillation (PDO), Magnuson et al. 1990; George et al. 2000; Livingstone and Dokulil 2001; Livingstone 2008). Over the middle and high latitudes of the Northern Hemisphere, the most prominent and recurrent pattern of atmospheric variability is the NAO index (Hurrell 1995). An impact of the winter NAO on thermal structure (water temperature, ice conditions) and biotic communities (e.g., spring plankton phenology) has been established in several European lakes (George et al. 2000; Straile 2000; Blenckner et al. 2007) and in the Baltic Sea (Alheit et al. 2005) and to some extent in the North Sea (Sharples et al. 2006). The NAO can, however, only adequately capture variability for parts of the year (mainly) due to the given movement of the NAO centres of action through the annual cycle (Hurrell et al. 2003). Thus, several studies indicated that water temperature and phenological events during spring and summer (Livingstone and Dokulil 2001) may not be related to the winter NAO, but instead correlate to warming trends during specific periods in spring (Gerten and Adrian 2000; Wagner and Benndorf 2007).

The identification of such crucial phases is, however, not at all trivial. Fixed periods or month (e.g., May, representing spring) have been used in correlative approaches focusing on specific systems (e.g., Gerten and Adrian 2000; George et al. 2000; Straile 2000; Arhonditsis et al. 2004). In lentic environments, this may be problematic since biotic characteristics depend on physical processes (e.g., ice-off, stratification), which have been clearly shifted as a consequence of climate warming (Gerten and Adrian 2000; Weyhenmeyer et al. 2008; Thackeray et al. 2010). Using fixed periods is also problematic in comparative studies across latitude or altitude (Livingstone and Dokulil 2001). Additionally, recent studies have shown that warming is not a continuous process, neither with respect to the seasonal pattern nor to the long-term trends (e.g., Gerten and Adrian 2001; Arhonditsis et al. 2004). Therefore, using a phenomenologically defined approach (e.g., phases of physical lake characteristics) might be more appropriate in this context than using fixed months. The identification of the timing of phenomenological phases requires simple markers (e.g., temperature thresholds) that are easy to determine. Since warming effects have been particularly pronounced during the transition from winter to spring and summer (Livingstone and Padisak 2007; Mooij et al. 2008; Adrian et al. 2009), it is necessary to focus on this period. In particular, however, to be useful in climate-related research, phases should be sensitively influenced by large-scale climatic drivers. A further precondition is that climate-sensitive phases are also ecologically sensitive, affecting biotic key processes and structures (compare Gerten and Adrian 2000; Sommer et al. 2007; Blenckner et al. 2007).

**Fig. 1:**
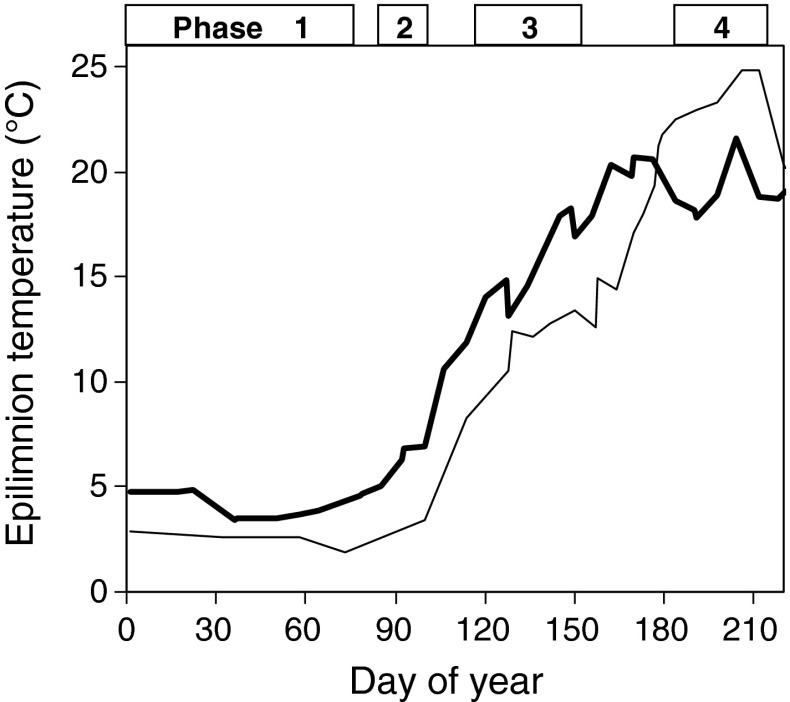
Pattern of epilimnetic water temperatures (3 m) from January to August in 2 years (2006 *light line*, 2007 *bold line*) of the long-term data set in Saidenbach Reservoir

What is missing as of yet, however, are classifications of sensitive phases for the screening of long-term data sets that have general applicability for a specific type of habitat (e.g., dimictic lakes in the temperate region) and that are well defined. The fundamental difficulty is the differentiation between warm and cold years since the same year can be colder during winter and spring, but warmer during summer compared to another year (Fig. 1). This example from the Saidenbach Reservoir illustrates that in order to be able to separate cold from warm patterns from long-term temperature records, one also needs phase-specific threshold temperatures defining warm and cold phases by a standardized procedure. A dual system using simple categories (warm/cold) for comparison of physical and biotic effects may accentuate differences between lakes or unveil general trends in the occurrence of seasonal sensitive phases.

Clearly, such a phenomenological approach, when used in a comparative analysis encompassing larger geographic scales, would have to take latitudinal gradients in seasonal timing into account (compare Weyhenmeyer et al. 2004). It is well established that changes in the global radiative heat balance correlating to global and regional climate change have an impact not only on the atmosphere and oceans, but also on water temperatures and stratification pattern of individual aquatic systems (Edinger et al. 1968; Sharples et al. 2006; Livingstone 2008). The main physical mechanisms that determine the heat balance of a lake involve one essentially astronomically determined variable (clear-sky solar radiation) and four meteorological variables (air temperature, cloud cover, wind speed and relative humidity) (Edinger et al. 1968; Arhonditsis et al. 2004). As the annual net radiation totals are increasing with decreasing latitude (Kondratjev 1969), we postulate that global irradiation can be employed for the identification of latitudinal gradients in the timing of sensitive phases of physical lake characteristics.

Here we present a concept that aims at a general applicability for the analysis of climate-induced changes in dimictic ecosystems. Our approach is based on an extensive data set of biotic and abiotic variables of a dimictic lake, the Saidenbach Reservoir (Germany) and is extended for dimictic lakes in Europe along a latitudinal gradient. Additionally, we applied the concept to examine the coherence among the dimictic reference lake and stations in the Baltic Sea and North Sea. Finally, we provide examples for the analysis of ecological responses to climate warming and for trend analysis with this approach. Specifically, we will:

1. Develop the approach based on the results of Saidenbach Reservoir
  - Define four phenomenological phases of physical lake characteristics.
  - Estimate threshold temperatures separating cold (C) from warm (W) phases.
  - Derive for each year of a long-term data set a specific CW-code indicating the temperature pattern within this year (e.g., phase 1 cold, phase 2 warm, etc.).
2. Extend the approach for the analysis of temperature patterns along a latitudinal gradient in Europe
  - Develop and validate a model to quantify the latitudinal shift in timing of sensitive phases by using cumulative global irradiation.
  - Validate thresholds estimated according to 1b for all sites.
  - Derive CW-codes analogous to step 1c for each study site and year.
  - Analyse cross-system coherence among lakes at different latitudes and coastal marine systems.
3. Apply CW-codes to analyse
  - Trends in the occurrence of patterns of warm phases.
  - Instantaneous or/and time-delayed responses of abiotic and biotic criteria as related to warming patterns during the phases.

With this phenomenological approach, we aim to provide a tool for a coherence-based analysis of dimictic aquatic systems, which may enlarge our insight into prediction of warming patterns and ecosystem response across latitudinal gradients.

## Methods

### Aquatic systems studied

In the present study, we estimate inter-annual differences in temperature patterns from six limnologically diverse dimictic lakes in Europe, and from five stations in the Baltic Sea and three stations in the North Sea. Some relevant physical and geographical characteristics of the study sites are listed in Table 1. The study sites are distributed at latitudes from 47 to 59°N and at longitudes from 6 to 18°E. Altitudes ranged from 0 m in coastal areas (Baltic and North Sea) to 439 m (Saidenbach Reservoir and Lake Greifensee) in mountainous areas. The surface areas of the lakes ranged from 1.5 (Saidenbach Reservoir) to 24 km^2^ (Lake Erken) at mean depths between 7.4 (Bautzen Reservoir) and 22.3 m (Lake Stechlinsee). The marine systems are characterized by larger surface areas and higher salinity compared to lakes. Salinity ranges from approximately 22 psu in the southeastern North Sea to more than 35 psu in the northwest, where oceanic Atlantic water enters the North Sea. In contrast to the North Sea, the Baltic Sea is a brackish sea with a unique, large estuarine basin (Hinrichsen et al. 2007). Surface salinity has remained fairly constant in the Baltic Sea since 1990, at around 8 psu from the Arkona Basin (between Sweden and Germany) to 16 psu in the Kiel Bight. Salinity changes abruptly below 30–60 m from the more fresh surface waters to the saltier deepwater (Eilola and Stigebrandt 1998). Apart from permanent haloclines, in the Baltic Sea, there is also a temperature layering with dimictic character (Schiewer 2008). Stronger solar heating during spring and summer results in thermal stratification of the water column leading typically to a 10–20-m-thick, warm mixed layer (Hinrichsen et al. 2007). Viewing the tides as providing a persistent ‘‘background’’ level of mixing, thermal stratification develops in early summer and then strengthens throughout the summer months also in the North Sea (Elliott and Clarke 1991; Sharples et al. 2006).

**Table 1:** General characteristics of the aquatic sites and the time period of the long-term monitoring programme

Site	Type	Location	Latitude (N)	Longitude (E)	Salinity (PSU)	Altitude (m)	Mean depth (m)	Surface area (km^2^)	Times series (years)	Time shift (days)
Greifensee^1^	Dimictic Lake	Switzerland	47°20′	8°40′		435	18.0	8.4	1998–2010	−7.1
Saidenbach^2^	Dimictic Reservoir	Germany	50°44′	13°14′		439	15.3	1.5	1975–2010	0.0
Bautzen^3^	Dimictic Reservoir	Germany	51°12′	14°27′		168	7.4	5.3	1977–1999	1.5
Scharmützelsee^4^	Dimictic Lake	Germany	52°15′	14°03′		38	8.9	12.1	1993–2010	3.8
Stechlinsee^5^	Dimictic Lake	Germany	53°09′	13°01′		60	22.3	4.5	1999–2008	5.8
Erken^6^	Dimictic Lake	Sweden	59°31′	18°35′		11	9.0	24.0	1988–2006	19.5
Oder Bank^7^	Baltic Sea	Germany	54°05′	14°10′	7	0	13.5	390,000	1998–2009	8.1
Kiel Bight^7^	Baltic Sea	Germany	54°30′	10°16′	16	0	13.5	390,000	1987–2010	8.9
Fehmarn Belt^7^	Baltic Sea	Germany	54°36′	11°09′	15	0	28	390,000	1988–2009	8.6
Darss Sill^7^	Baltic Sea	Germany	54°70′	12°69′	10	0	21	390,000	1995–2009	9.4
Arkona Basin^7^	Baltic Sea	Germany	54°88′	13°87′	8	0	45	390,000	2003–2009	9.8
Ems^7^	North Sea	Germany	54°17′	6°35′	22	0	33	575,000	1990–2009	8.2
German Bight^7^	North Sea	Germany	54°17′	7°45′	33	0	33	575,000	1990–2009	8.2
North Sea Buoy^7^	North Sea	Germany	55°00′	6°20′	32	0	42	575,000	1991–2009	9.3

The last column gives the latitudinal time shift in the start of sensitive phases in relation to timing in Saidenbach Reservoir

For more details to the study sites see *1* Bürgi et al. (2003), *2* Rolinski et al. (2007); Horn and Horn (2008), *3* Benndorf et al. (2001), *4* Grüneberg et al. (2011), *5* Mehner et al. (2008), *6* Pettersson et al. (2003), *7* http://www.bsh.de/de/Meeresdaten/Beobachtungen/MARNET-Messnetz/index.jsp

Saidenbach Reservoir is used as the reference lake of our study as in this system, the CW-concept was developed first (Wagner et al. in press). Long-term meteorological, hydrological, and limnological data sets covering 35 years and the detailed knowledge of the food web structure (Horn and Horn 2008) reveal best preconditions to develop the above described concept.

### Data pool

In Saidenbach Reservoir, vertical profiles of water temperature were recorded at 1-m intervals with a digital probe usually at weekly intervals (Horn et al. 2011; Wagner et al. in press; L. Paul, unpublished data). Additionally, we used temperature data from quasi-continuous measurements (hourly) from 2005 to 2009. Temperature data were determined with a thermistor probe every 2 weeks in Bautzen Reservoir (Wagner and Benndorf 2007). Depth profiles of temperature were measured above the deepest point of the southern main basin of Lake Scharmützelsee with a multiparameter probe (Grüneberg et al. 2011; personal communication J. Rücker from BTU Cottbus) and biweekly (partly weekly) close to the deepest point of the oligotrophic Lake Stechlinsee (Mehner et al. 2008; personal communication P. Kasprzak (IGB Berlin)). For Lake Erken (Pettersson et al. 2003), data series of daily measurements of temperature were provided by G. Weyhenmeyer (Uppsala University, personal communication). For Lake Greifensee (Livingstone and Lotter 1998; Bürgi et al. 2003; Franssen and Scherrer 2008), we combined water temperature data measured hourly using an automatic hydro-meteorological station (2006–2010; http://club.swiss-sailing.ch/greifensee/) and thermistor probes (1998–2010, http://www.awel.zh.ch/internet/baudirektion/awel/de/wasserwirtschaft/messdaten/see_qualitaet.html). Water temperature data from Baltic Sea and North Sea are based on quasi-continuous measurements at fixed monitoring stations (Table 1) being a part of an automated monitoring network (MARNET, http://www.bsh.de/de/Meeresdaten/Beobachtungen/MARNET-Messnetz) operated by the Federal Maritime and Hydrographic Agency of Germany (personal communication F. Nast and A.-C. Bohnenstengel (MARNET)). Additionally, we used temperature data measured weekly in Kiel Bight nearby the GEOMAR (http://www.geomar.de/service/wetter/, personal communication C. Clemmensen (IFM GEOMAR)). The site-specific duration of the time series of water temperatures covered by our investigation are shown in Table 1.

For the purpose of our study, we selected water temperatures measured at a depth horizon of 3 m corresponding to the illuminated, warm, wind-mixed layer that represents the epilimnion in lakes or the mixed layer in marine systems (Livingstone and Lotter 1998; Livingstone 2003; Schiewer 2008). Epilimnetic water temperatures are highly correlated with regional-scale air temperatures and show relatively low daily fluctuations (Livingstone and Dokulil 2001; Uhlmann et al. 2011). They control the metabolism and growth rates of many organisms and therefore may evoke a rapid and direct response of aquatic organisms, but also of entire ecosystems to climatic forcing (Edwards and Richardson 2004; Adrian et al. 2006; Wagner and Benndorf 2007). Weekly or biweekly field measurements of water temperatures were linearly interpolated, resulting in time series of daily water temperatures, respectively.

### Develop the approach based on the results of Saidenbach Reservoir

#### Step 1a: Define four phenomenological phases of physical lake characteristics

Acknowledging the strong relationship between climatic conditions and the thermal structure of dimictic aquatic systems, we selected four phenomenologically defined phases of physical characteristics (Uhlmann et al. 2011): the periods of inverse stratification during winter, spring overturn, early thermal stratification and summer stagnation (Table 2). These phases are considered to respond sensitive to climate warming (Benndorf et al. 2001; Livingstone 2003) but also critically influence phenology of key components of aquatic food webs (Adrian et al. 2006; Sommer et al. 2007; Blenckner et al. 2007). Therefore, the term sensitive phase means both climate-sensitive and ecological-sensitive. In temperate systems, the timing of winter stagnation (phase 1) depends on the establishment and duration of the ice coverage (Adrian et al. 1999; Rolinski et al. 2007; Weyhenmeyer et al. 2008) or (if no ice cover) on air and epilimnion temperatures (Hülsmann et al. in press). Following the melting of ice cover and warming of surface water, vigorous vertical mixing induced by wind and surface warming to 4 °C results in homothermy (Livingstone 2003) corresponding to the start of spring overturn (phase 2). Phase 2 is represented by the first day with 4 °C-homothermy in the water depths from 3 m to 10/15 m. We consider early thermal stratification (phase 3) to start when warming of epilimnion water exceeds 10 °C (Rolinski et al. 2007). During summer stagnation, dimictic systems are characterized by a stable thermal stratification with a warm epilimnion and often a successive increase in thickness of the epilimnion layer. As in Europe lake surface waters are typically at their warmest in July or early August (Arvola et al. 2010), phase 4 is fixed to July, without defining a threshold temperature for the start. The criteria used to define the start or end of the sensitive phases are summarized in Table 2. For each year of our reference period (1995–2009), we determined the respective day of the year of start (phase 2 and 3) or end (phase 1) of these phases as well as a mean date.

**Table 2:** Four phenomenologically defined phases of physical lake characteristics and criteria to determine the start/end of these phases in dimictic temperate systems

Phases	Criteria determining start/end	Time period day of year^a^	Mean temperatures during phases (°C)^a^
1. Inverse stratification (winter)	End at ice-off or* T*_3m_ > 3 °C	From day 01 to 71 (±27.2)	2.90 ± 0.61
2. Spring overturn	Start when* T*_0–10m_ = 4 °C	Day 91 (±13.3)	4.04 ± 0.67
3. Early thermal stratification	Start when* T*_3m_ > 10 °C	From day 121 (±7.6) to 151	13.89 ± 1.89
4. Summer stagnation	Start 1 July	From day 182 to 212	19.94 ± 1.69

For a given time period within the sensitive phases (column 3), mean epilimnion temperatures (±SD) were calculated (column 4) from daily measured or interpolated time series of water temperature data (1990–2009) in Saidenbach Reservoir

^a^As related to our reference lake (Saidenbach Reservoir)

For the reference lake (Saidenbach Reservoir), besides water temperature, we analysed climatic variables (i.e., air temperature, irradiance, precipitation, wind speed) measured daily (1999–2010) at a meteorological station located approximately 4 km from the study site (Forchheim, 50.71°N, 13.27°E, 563 m, http://www.landwirtschaft.sachsen.de/Wetter09). To examine potential relationships between macroscale atmospheric processes and thermal properties in Saidenbach Reservoir, the winter (December–March) index of the NAO was taken from http://www.cgd.ucar.edu/cas/jhurrell/indices.html. Spearman rank order correlations were used to evaluate the relationship between these climatic parameters and the epilimnion (3 m depth) temperatures during the four sensitive phases in Saidenbach Reservoir.

#### Step 1b: Estimate threshold temperatures separating cold from warm phases

We relied on natural climatic year-to-year variability for water temperatures to separate cold from warm phases. Average temperatures for the four phases were calculated on the basis of the daily measured or interpolated time series of epilimnion temperature (phases 1, 3 and 4) and 10-m temperature (phase 2) for every year as time-weighted means of temperatures, respectively. According to the days of year, which are defined by step 1a for the start and end of the four phases in Saidenbach Reservoir, we considered the time period from 1 January to 15 March for phase 1 and water temperatures determined on 1 April for phase 2. To characterize phases 3 and 4, mean epilimnion temperatures were estimated as time-weighted average over the 31 days that followed 1 May and 1 July, respectively. The threshold values were estimated from averages of phase-specific water temperatures during the last two decades (1990 through 2009) rounded to the integer. The time period after 1990 was selected as it represented a period with a consistent warming trend (Rolinski et al. 2007; Bates et al. 2008).

#### Step 1c: Derive CW-codes for each year of a long-term data set

Based on threshold temperatures defined in step 1b (see also Table 2), we classified phases of a year as cold when time-weighted means of long-term temperature data determined as described in step 1b fell below the threshold or as warm when threshold temperature was equalled or exceeded, resulting in a simple code for each year (e.g., phase 1 warm, phase 2 warm, phase 3 warm, phase 4 cold = WWWC, see 2007 in Fig. 1).

### Extend the approach to dimictic aquatic systems along a latitudinal gradient

#### Step 2a: Develop and validate a model to quantify the latitudinal shift in timing of sensitive phases by using cumulative global irradiation

Global irradiance may control the energy balance of a lake varying with latitude, season, cloud coverage, atmospheric pollution and solar altitude (Edinger et al. 1968; Arhonditsis et al. 2004; Bluszcz et al. 2008). In our empirical model, global irradiation has a threefold role. Firstly, as a causal variable, it is involved explicitly in processes that determine epilimnion temperature directly. Secondly, it is autocorrelated with the other meteorological variables (wind speed, cloud cover and air temperature) that co-determine lake temperature. Thirdly, global irradiance may control the timing (day of year) when a sum of the global radiation is exceeded for given latitudes. Solar irradiance data are collected at local meteorological stations by ground based pyranometers (see step 1a) and by orbiting satellites (NASA and other satellites). These data are easily available from 1978 to the present on the internet (e.g., World Radiation Data Centre, http://wrdc-mgo.nrel.gov/). To compare different latitudes (lat), we used a heuristic algorithm, based on the total sum of the maximal (i.e., cloudless) solar radiation (*G*_max,lat,*t*_) during the time period between 1 January (day 1) and a given date (*t*):

1 $$ G_{{\text{max,\,lat}}, t} = \sum\limits_{1}^{t} {G_{{\text{max,\,lat}}}} $$

where *G*_max,lat_ is the theoretical daily sum of the global radiation for a given latitude and cloudless sky, but respecting an empirical turbidity coefficient of the atmosphere (*T*_*L*_ = *2*). The daily sum (*G*_max,lat_ in kJ cm^−2^) was calculated by using common astronomical formulae according to the standard procedure of the Society of German Engineers (VDI 3789-part 2 1994); similar formulae can be found elsewhere, for example, in Walsby (1997). The results of the formula were validated against publicly available long-term data from the World Radiation Data Centre. Based on this, a given date (here: start of sensitive phases of physical lake characteristics) can be transferred from one latitude (lat1) to another (lat2) by using the following algorithm:

1. Select a specific date characteristic of a sensitive phase (e.g., 1 May = day 121, representing the mean start of early stratification in the reference system),
2. Calculate the total sum of the global radiation (*G*_max,lat1,121_) for the time period between day 1 and day 121 for this latitude.
3. Find the day *t* where *G*_max,lat1,121_ = *G*_max,lat2,*t*_, that is, the day with the same cumulative radiation since 1 January for another latitude.

The algorithm is intentionally simple using only astronomical relationships, therefore further generalizations will be necessary for an application to other regions of the world, for lakes at higher altitudes or for regions with very strong continentality (Hela 1953). The algorithm was applied in the present study to define the latitudinal shifts in the beginning of the second to fourth phase.

To validate latitudinal shifts in the start of phases estimated by the *G*_max,lat_-algorithm, we used the day of year of the beginning of the respective phase that is determined by applying temperature criteria (second column in Table 2) to all lake sites. Using linear regressions, we tested exemplarily the effect of latitude on the time shift of the start of phases 2 and 3. Slope coefficients of linear regressions quantified rates of change in timing of sensitive phases expressed as days per one degree of latitude. We used paired *t* test analysis to compare the latitudinal shift in timing of phases determined by *G*_max,lat_-algorithm and by water temperature criteria, respectively (all data were approximately normally distributed).

#### Step 2b and 2c: Validate temperature thresholds for other study sites and derive CW-code

After determining the latitudinal shift in the start of the sensitive phases as related to timing in our reference lake (Tables 1, 2), steps 1b and 1c are repeated for every site given in Table 1 and every year of the long-term data sets.

#### Step 2d: Analyse cross-system coherence among lakes at different latitudes and coastal marine systems

We defined spatial coherence as the degree of similarity between time series of CW-codes determined simultaneously in our reference lake and another aquatic system. The concept of coherence in this context is not well defined in a mathematical sense (Livingstone et al. 2010). The degree of similarity was estimated as the relative portion of years of the time-series (expressed as %) in which the CW-codes in the respective study site equalled the classification determined in the reference lake (Saidenbach Reservoir). Additionally, the mean square contingency coefficient Phi and the Odds ratio for the 2 × 2 contingency tables of matching and non-matching classifications were used to discover the strength of agreement. The Phi coefficient is an equivalent to the Pearson correlation for binary data, and the Odds ratio the quotient of the products of the number of matching $\left( {N_{\text{cc}} \cdot N_{\text{ww}} } \right)$ and non-matching $\left( {N_{\text{cw}} \cdot N_{\text{wc}} } \right)$ cases: ${\text{Odds}} = \frac{{N_{\text{cc}} \cdot N_{\text{ww}} }}{{N_{\text{cw}} \cdot N_{\text{wc}} }}$.

In a first step, relative similarities among limnetic and marine systems and the reference lake were estimated for the CW-codes determined by using constant (no site specific) temperature thresholds derived from lakes (phase 1: 3 °C, phase 2: 4 °C, phase 3 14 °C, phase 4: 20 °C). In a second step, the degree of similarity among the reference lake, the five stations in the Baltic Sea and the three stations in the North Sea (Table 1) was estimated for CW-codes derived from site-specific temperature thresholds that are defined as average (±SD) over the stations investigated in the Baltic Sea and the North Sea, respectively.

### Application of CW-codes

#### Step 3a: Analyse trends in the occurrence of patterns of warm phases

The CW-codes determined for the long-term data of our reference lake (Saidenbach Reservoir) were used to analyse exemplarily trends in the occurrence of code patterns since 1975. Trend curves for the probability of warm phases were fitted by using a generalized linear model (*GLM*) with binomial distribution and logit link (Venables and Ripley 2002). The overall trend of the number of warm periods per year was tested by the Mann–Kendall trend test (Hipel and McLeod 2005) implemented in R package Kendall (McLeod 2011).

#### Step 3b: Analyse responses of abiotic and biotic criteria to phase-specific patterns of warming

Acknowledging that differences in climate warming between seasons, years and latitudes will influence its effect on organisms according to their life cycle, CW-codes can be used to analyse and subsequently predict direct and indirect responses of abiotic and biotic criteria to warming. As an example, we tested the effect of current CW-code patterns during the four sensitive phases on the stability frequency in the upper 15 m of the water column in Saidenbach Reservoir during July (1988–2009, L. Paul, unpublished data). The Brunt--Väisälä frequency indicating the stability of temperature stratification was calculated from vertical density gradients derived from water temperatures (Uhlmann et al. 2011). Secondly, we analysed whether climate-sensitive phases are considered to be also ecologically sensitive by estimating the effect of cold--warm pattern on the population dynamics of a key plankton species (*Daphnia*) in Saidenbach Reservoir during August (1998–2009). *Daphnia* biomass was taken from Wagner et al. (in press). To examine whether the mean stability frequency of the water column or the biomass of *Daphnia* were related to instantaneous or rather to delayed effects of warming during the four phases, respectively, we used a *U* test for unequal sample sizes after testing for homogeneity of variances (Levene test). In general, probabilities <0.05 were considered to indicate a significant relationship. Statistical analyses were conducted with SigmaPlot 11.0 **(**Systat Software, Inc. 2008) and with the R system (R Development Core Team 2011).

## Results

### Develop the approach from long-term data of Saidenbach Reservoir

**Fig. 2:**
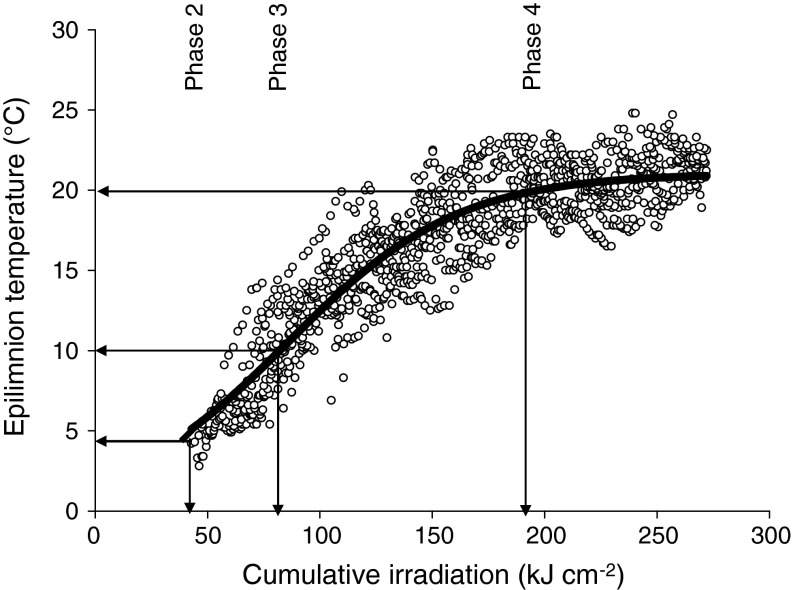
Regression analysis of daily irradiation cumulated from 1 January until a given day (cumRad) and the corresponding epilimnion temperature (*T*_epi_) in Saidenbach Reservoir (from April to August 1999–2009, *N* = 1,254, *T*_epi_ = 21.048/(1 + exp(−(cumRad −85.84)/37.33)), *r* ^2^ = 0.85, *P* < 0.0001). The *arrows* describe the mean cumulative irradiation when temperature criteria from Table 2 defining the start of phases are equalled

Pooling data of 11 years (1999–2009, Fig. 2) revealed a significant effect of cumulative irradiation on the epilimnetic temperatures in Saidenbach Reservoir accounting for about 85 % of the temperature variability. We determined a sigmoidal dependency between the irradiation cumulated from 1 January through a given day and the corresponding epilimnetic water temperature. Cumulative irradiation exceeding a critical value of 32 kJ cm^−2^ corresponded to the start of spring overturn (phase 2) when warming to 4 °C results in homothermy (Fig. 2). A further increase in cumulative irradiation to 83 kJ cm^−2^ coincided with the start of early thermal stratification (phase 3) with epilimnion temperatures exceeding 10 °C. During the period of summer stagnation (phase 4), the increase in epilimnion temperatures asymptotically approaches a saturation when cumulative irradiation exceeds about 197 kJ cm^−2^ (phase 4). Epilimnion temperatures in Saidenbach Reservoir correlated significantly to the monthly means of irradiation (*c*_sp_ = 0.27, *P* < 0.0001), wind speed (*c*_sp_ = −0.29, *P* = 0.0041) and air temperature (*c*_sp_ = 0.78, *P* < 0.0001) and weaker to precipitation (*c*_sp_ = 0.08, *P* < 0.0001); however, cumulative irradiation was the best predictor for epilimnion temperatures (*c*_sp_ = 0.91, *P* < 0.0001).

When testing for relations between climatic variables and water temperature during sensitive phases, significant correlations were revealed with air temperature and cumulative irradiation for all phases (Table 3). By contrast, years with a high NAO index were significantly correlated only with temperatures in phase 1 and 2, but not in phase 3 and 4.

**Table 3:** Pearson product moment correlation coefficients to determine the influence of climatic forcing (NAO index, air temperature, cumulative irradiation) on epilimnion temperatures during sensitive phases (see Table 2) in Saidenbach Reservoir, 1999–2009

Phase	1 (ice-off)	2	3	4
NAO index	−0.615*	0.661*	ns	ns
Air temperature	−0.748*	0.685*	0.717*	0.733*
Cum. irradiation	0.584*	0.721*	0.685*	0.745*

*ns* not significant,* NAO* North Atlantic oscillation

* *P* < 0.05

**Fig. 3:**
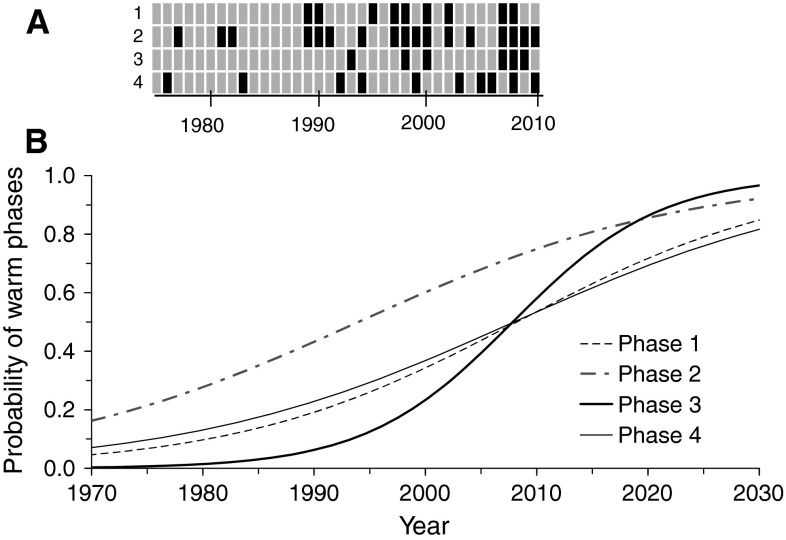
The grid chart of yearly CW-codes visualizes the pattern of warm (*black*) and cold (*light grey*) sensitive phases (numbers 1–4 on *y* axis) during the last 35 years in Saidenbach Reservoir (**a**). The lower graph (**b**) quantifies the trend in the probability of occurrence of warm phases since 1970 and provides a prediction for 2030. Trends are described by logistic equations *y* = exp(a + b · x)/(1 + exp(a + b · x) fitted by a generalized linear model for binomial data (GLM): *phase 1* *a* = −158.53, *b* = 0.07894, dev = 3.69, *P* = 0.055; *phase 2* *a* = −136.29, *b* = 0.06835, dev = 3.77, *P* = 0.052; *phase 3*: *a* = −304.15, *b* = 0.15148, dev = 7.35, *P* = 0.007; *phase 4* *a* = −135.92, *b* = 0.06769, dev = 3.00, *P* = 0.083. Reduction of residual deviance (dev) is compared to the null model with no trend. The overall trend of all phases is significant with τ = 0.428, 2-sided *P* = 0.0011 (Mann–Kendall trend test)

In our reference lake (Saidenbach Reservoir) ice coverage during phase 1 is terminated on average on 10 March followed by spring overturn (phase 2), which started on average on 1 April (Table 2). Thermal stratification (phase 3) started on average on 30 April. The water column was strictly stratified during July (01–31, phase 4) in all years of the long-term data set. Mean water temperatures (1990–2009) during the sensitive phases amounted to 2.9 °C (phase 1), 4.0 °C (phase 2), 13.9 °C (phase 3) and 19.9 °C (phase 4) in Saidenbach Reservoir. Based on mean values rounded to the integer (Table 4), phases of a year were classified for the long-term data set (1975–2010), and yearly CW-codes were visualized in a grid chart (Fig. 3a). According to temperature patterns in Fig. 1, the code for 2006 is CCCW and for 2007 WWWC.

### Extend the approach for the analysis of temperature patterns along a latitudinal gradient in Europe

**Fig. 4:**
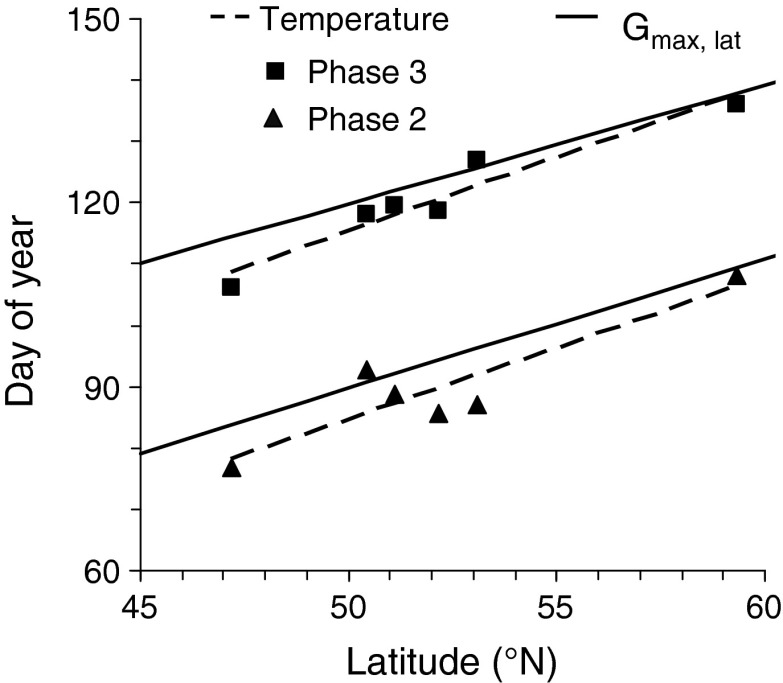
Regression analysis between latitudes (lat) of the lakes studied (see Table 1) and the day of year (Julian day) when sensitive phases 2 and 3 started. For definition of phases see Table 2. Timing was determined by threshold temperatures (*T*) documented in Table 2 (*dashed lines*) and by the *G*_max,lat_-algorithm (Eq. 1) (*solid lines*). doy_phase 2, *T*_ = (2.33 · lat) −31.5, *N* = 6, *r* ^2^ = 0.76, *P* = 0.025; doy_phase 3, *T*_ = (2.35 · lat) −1.46, *N* = 6, *r* ^2^ = 0.93, *P* < 0.001; doy_phase 2,_ *G*_max,lat_ = (2.125 · lat) −16.57; doy_phase 3,_ *G*_max,lat_ = (1.97 · lat) +21.74

Linear regression analysis revealed a significant effect of latitude of study sites on the day of the year at which phases 2 and 3 started, no matter whether timing was determined by threshold temperatures or by the *G*_max,lat_-method (Fig. 4). In general, using the *G*_max,lat_-algorithm that transfers the beginning of a sensitive phase from one latitude to another resulted in a slightly later start of the phases compared to the day of year derived directly from water temperatures according to criteria in Table 2. Rates of changes in timing of sensitive phase amounted to 2.3 days per one degree of latitude using temperature thresholds and 2.1 days per one degree of latitude using *G*_max,lat_. Paired *t* test revealed, however, no significant differences in timing between dates determined by *G*_max,lat_ or temperature criteria both for the start of spring overturn (*t*_6_ = −2.202, *N* = 6, *P* = 0.09) and of early thermal stratification (*t*_6_ = −2.337, *N* = 6, *P* = 0.06) indicating that the *G*_max,lat_-method accurately reproduced the timing of the phases. To estimate the latitudinal time shift in the start of the sensitive phases for further analysis, we averaged slopes of all linear regressions in Fig. 4 resulting in a mean time shift of 2.19 (±0.15) days per one degree of latitude. Within the latitudinal gradient covered by lakes of our study (47°–59°N), the maximum shift in the start of sensitive phases as related to our reference lake (Saidenbach Reservoir) amounted to +19 days (Lake Erken) and −7 days (Lake Greifensee), while the time shift did not exceed 6 days for German lakes and ranged between 8 and 9.5 days for the marine systems considered here (last column in Table 1).

**Fig. 5:**
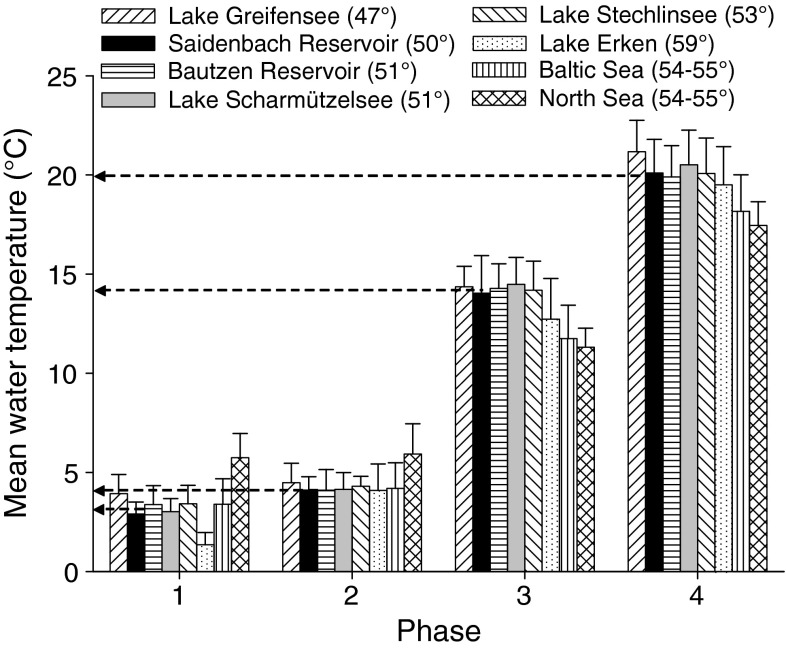
Mean values and SD of water temperatures during the four sensitive phases (see Table 2) from temperature data for the last two decades (1990–2009) for lakes within a latitudinal gradient from 47 to 59°N (considering the time shift with respect to Saidenbach Reservoir as given in Table 1), the five stations in the Baltic Sea and the three stations in the North Sea. *Arrows* mark mean values for all lake systems. For definitions of phases 1–4 see Table 2

After accounting for latitudinal shifts in timing, mean water temperatures of sensitive phases were quite similar between lakes within a latitudinal gradient from 47 to 54°N (Fig. 5). In Lake Erken (59°N) mean epilimnetic temperatures were lower compared to the other lakes during phase 1 and 2 (Fig. 5). Mean temperatures of the mixed layer determined in the Baltic Sea matched values determined in lakes for phase 1 and 2, but were lower for the subsequent phases (early thermal stratification and summer stagnation). Temperatures observed at stations in the North Sea were higher during winter and spring overturn, but fell below values determined in other systems during early thermal stratification and summer (Fig. 5).

**Fig. 6:**
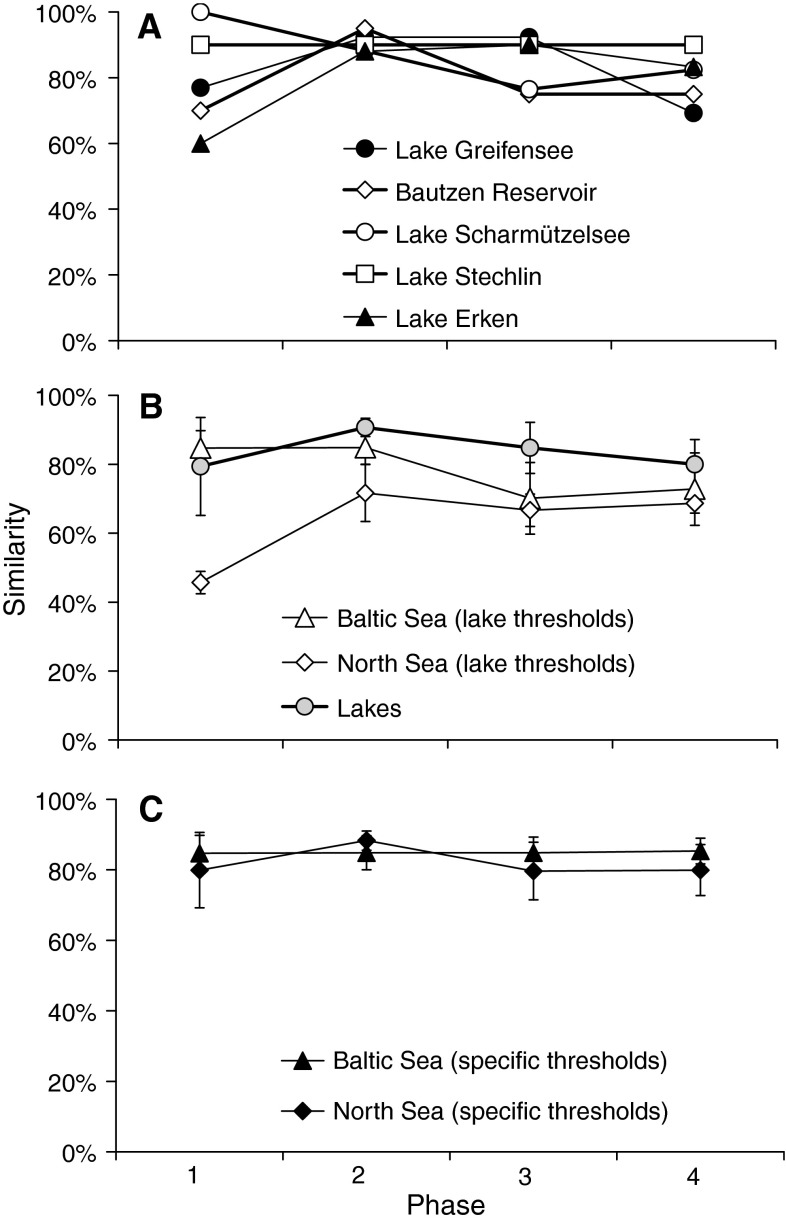
Relative similarity (mean ± SD of all years and study sites) between CW-codes of the reference lake (Saidenbach Reservoir) and CW-codes of lakes within a latitudinal gradient from 47°N to 59°N (**a**, **b**), five stations in the Baltic Sea and three stations in the North Sea using constant temperature thresholds derived for lake sites (**b**) and marine systems determined by using site-specific temperature thresholds for separating cold from warm phases (**c**). For definitions of phases 1–4 see Table 2

By using a constant temperature threshold (derived from lakes, Table 4) for each of the aquatic systems to classify the respective phase as warm or cold, all relationships between the reference lake and the other limnetic systems were highly significant at *P* < 0.001 (Table 5, Fisher’s exact test). Similarity to the reference lake ranged for single phases from 79 % or Phi = 0.56 (Lake Erken, Bautzen Reservoir) to 90 % or Phi = 0.80 (Lake Stechlinsee) or even to 100 % (winter stagnation in Lake Scharmützelsee) (Table 5, Fig. 6a). The mean similarity over all phases amounted to 84 % between the reference lake and all other lakes along the latitudinal gradient from 47 to 59°N. If we compare seasonal coherence, highest similarity among lakes was determined for the spring overturn (91 %) followed by early thermal stratification (85 %). However, some lakes deviated somewhat from the regional norm. For the shallower Bautzen Reservoir (Table 1), we found a high spatial coherence to our reference lake during spring overturn but relatively low coherence (70–75 %) during winter and during thermal stratification (Fig. 6a). During winter, coherence was also less pronounced between Saidenbach Reservoir and Lake Erken (the lake with the highest latitude), but despite the high latitude, the relative similarity increased to nearly 90 % during spring overturn and early thermal stratification (Fig. 6a). Lake Greifensee (lowest latitude) exhibited a mean similarity of warm--cold pattern over all phases as related to the reference lake of 82 % with lowest values during phase 4 (summer stagnation, 69 %).

**Table 4:** Sensitive phases of physical characteristics in dimictic temperate aquatic systems and temperature thresholds used to separate warm (≥) from cold (<) sensitive phases specified for lakes, the Baltic and the North Sea

Phases	Temperature thresholds determining warm phases in		
	Lakes (°C)	Baltic Sea (°C)	North Sea (°C)
1. Inverse winter stratification	3	3.0	5.5
2. Spring overturn	4	4.0	5.8
3. Early thermal stratification	14	11.5	10.8
4. Summer stagnation	20	18.3	17.0

Temperature thresholds are derived from average temperatures in the epilimnetic or mixed layer (phase 1, 3 and 4) or at a depth of 10–15 m (phase 2) according to Fig. 5

**Table 5:** Comparison of phase classifications of each study site (by using *constant* temperature thresholds derived from lakes (Table 4) to separate cold (C) from warm (W) phases) with CW-codes of the reference lake (Saidenbach Reservoir) by 2 × 2 tables with contingency coefficient Phi and Odds ratio

	Coherence		No coherence		Phi	Odds ratio	*P* value (Fisher’s exact test)
Prediction	C	W	C	W			
Observation	C	W	W	C			
Lake Greifensee	18	25	9	0	0.70	∞	<0.001
Bautzen Reservoir	46	17	15	2	0.56	24.8	<0.001
Lake Scharmützelsee	29	30	9	0	0.77	∞	<0.001
Lake Stechlinsee	19	17	3	1	0.80	86.2	<0.001
Lake Erken	43	15	1	13	0.60	46.6	<0.001
Oder Bank	18	10	2	7	0.52	11.85	0.004
Kiel Bight	53	29	6	8	0.69	30.20	<0.001
Fehmarn Belt	27	12	4	7	0.52	10.86	<0.001
Darss Sill	31	15	1	11	0.61	39.31	<0.001
Arkona Basin	14	6	1	7	0.45	10.93	0.029
Ems	26	20	18	11	0.23	2.59	0.061
German Bight	27	17	17	11	0.22	2.42	0.091
North Sea Buoy	16	13	7	6	0.38	4.75	0.029

*P* values are derived with Fisher’s exact test of the 2 × 2 table

If we compare the CW-codes determined in the marine systems with our dimictic reference lake using temperature thresholds derived for lake sites (Table 4), relationships to all stations in the Baltic Sea were significant at *P* < 0.05 (Table 5, Fisher’s exact test). Similarity in the cold--warm pattern between lakes and the Baltic Sea amounted to 78 % on average and even 85 % (ranging from 77 to 93 % for the five stations) for winter and spring overturn, and decreased, however, to less than 70 % during early thermal stratification and summer (Fig. 6b). By contrast, CW-codes determined for three stations in the North Sea generally deviated somewhat from the regional coherence (odds ratio between 2.42 and 4.75). Fisher’s exact test was not significant for the stations Ems and German Bight (Table 5) and warm--cold patterns mismatched by at least 46–68 % with the codes of the reference lake with the only exception of the spring overturn when similarity amounted to 72 % (Fig. 6b).

In a second analysis, we used site-specific temperature thresholds to classify the respective phase as cold or warm (Table 4) in the Baltic Sea and North Sea. As a consequence, the predictability of the cold--warm pattern was improved for the Baltic Sea and the North Sea (Fig. 6c) compared to similarities observed by using temperature thresholds from lakes. Spatial coherence between our reference lake and the marine systems was now significant (all *P* values <0.001, Table 6); the Odds ratio increased considerably for all stations in the North Sea and most stations (Fehmarn Belt, Arkona Basin, Darss Sill) in the Baltic Sea. The relative degree of coherence to the reference lake increased to 85 % (Baltic Sea), and 82 % for North Sea as mean values were averaged over all phases (Table 6, Fig. 6c).

**Table 6:** Comparison of phase classifications of marine study sites (all phases) by using *specific* temperature thresholds for North Sea and Baltic Sea (Table 4) to separate cold (C) from warm (W) phases with CW-codes of the reference lake (Saidenbach Reservoir) by 2 × 2 tables with contingency coefficient Phi and Odds ratio

	Coherence		No coherence		Phi	Odds ratio	*P* value (Fisher’s exact test)
Prediction	C	W	C	W			
Observation	C	W	W	C			
Oder Bank	15	14	5	3	0.57	12.77	<0.001
Kiel Bight	46	34	13	3	0.68	37.99	<0.001
Fehmarn Belt	24	18	6	0	0.77	∞	<0.001
Darss Sill	31	20	1	6	0.76	91.05	<0.001
Arkona Basin	14	10	1	3	0.72	37.76	<0.001
Ems	29	28	15	3	0.56	17.26	<0.001
German Bight	35	25	9	2	0.70	44.91	<0.001
North Sea Buoy	19	17	4	2	0.72	35.08	<0.001

*P* values are derived with Fisher’s exact test of the 2 × 2 table

Pooling the data of all systems and years revealed no significant effect of latitude, salinity, altitude, mean depth or surface area on the similarity of the cold--warm pattern (which was based on constant temperature thresholds for the lakes within the latitudinal gradient from 47 to 59°N but specific temperature threshold values for Baltic Sea and North Sea) among the systems.

### Apply CW-codes to analyse trends and responses of abiotic and biotic criteria

As exemplified for our reference lake, the grid chart of yearly CW-codes (Fig. 3a) visualized the pattern of warm and cold phases indicating clearly an increase of the occurrence of warm phases during the period under investigation (1975–2010). Four warm phases per annum occurred for the first time in 2008 (Fig. 3a). The probability of warm phases shows a sigmoidal trend for all four sensitive phases (GLM fits in Fig. 3b) and though not strictly significant for all individual sensitive phases because of still too small sample size, clearly a general overall pattern emerges. According to this, a Mann–Kendall trend test for the number of warm phases per year was highly significant (τ = 0.428, *P* = 0.0011). The probability of warm phases during early thermal stratification shows a significant warming trend (*P* = 0.007) from 0 to 58 % for the period from 1975 to 2010 (Fig. 3b). Our results allow a prediction of the near-future increase in the probability of warm phases amounting to about 97 % (2030) for early thermal stratification.

**Fig. 7:**
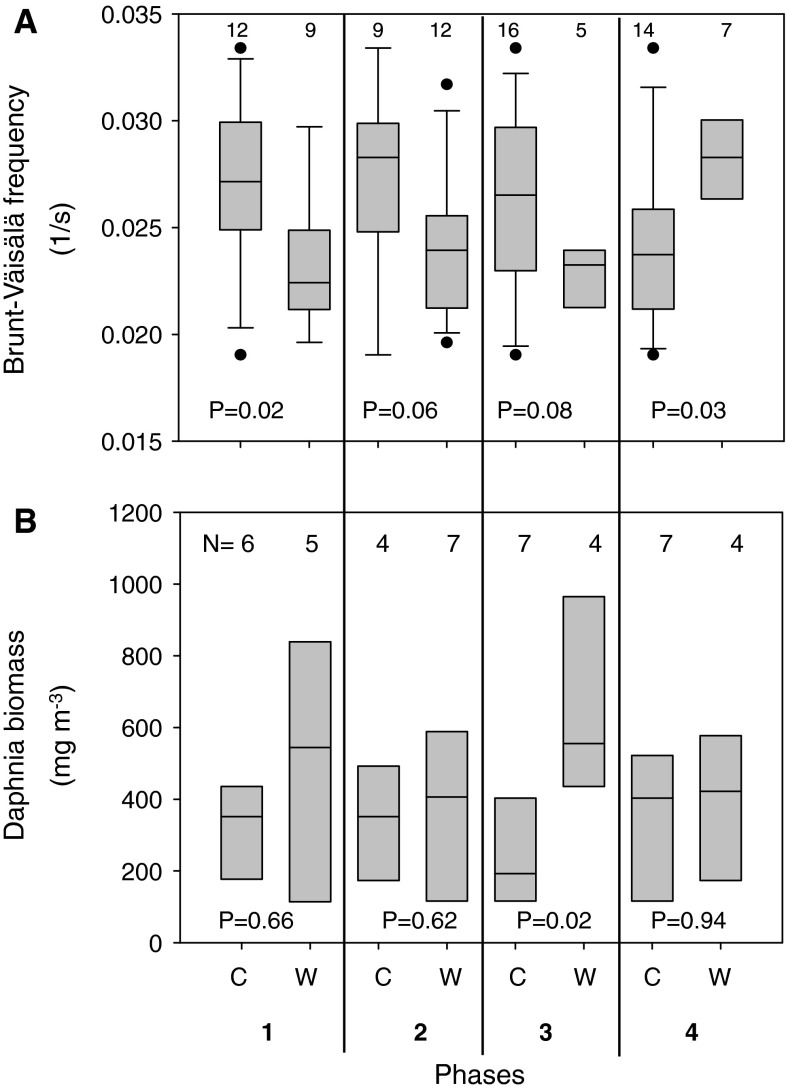
*Box* *plots* (median, 5, 25, 75 and 95th percentiles) of mean stability frequencies (after Brunt--Väisälä) of the upper 15 m during July (1988–2009) (**a**) and biomass of *Daphnia* during August (**b**) in Saidenbach Reservoir (1998–2009), subdivided into years with cold (C) and warm (W) conditions during the four sensitive phases according to the CW-codes determined yearly. *N* corresponds to the number of years. For definitions of phases 1–4 see Table 2

Examples how to apply the CW-code for analysing abiotic and biotic responses to climate variability are shown in Fig. 7. Separating our data set of Brunt--Väisälä frequencies according to the classification of the phases as warm or cold revealed that the epilimnion temperature during winter and summer stagnation had a significant effect on the stability frequency of the upper 15 m of the water column during July (Fig. 7a). On the contrary, the mean stability frequency did not depend significantly on warming during spring overturn or early thermal stratification. A high stability frequency (>0.025) during July can be expected under cold conditions during winter and warm conditions during summer stagnation. Applying the same approach for long-term data of *Daphnia* biomass during August (1998–2009), we found that moderate warming during early thermal stratification rather than winter or summer temperatures had a significant effect on its population dynamics during summer. Warming exceeding a critical temperature (14 °C) during early thermal stratification increased the stability of the *Daphnia* population during summer in Saidenbach Reservoir (Fig. 7b).

## Discussion

### Phenomenological phases of physical lake characteristics defined by water temperatures and cumulative irradiation

Our approach provides a phenomenology-based categorical analysis for comparing changes in cold--warm patterns of climate-sensitive phases and the coherence in the response between dimictic aquatic ecosystems along a latitudinal gradient. We found a high degree of spatial and temporal coherence of cold--warm patterns during sensitive phases for all lakes and the Baltic Sea, which is based on two preconditions met by our concept: First, our approach used four phenomenological phases well defined by physical characteristics (Table 2) as period of reference instead of fixed periods or whole years to realize a general applicability of the approach for dimictic systems in the temperate region. Second, our results verified that timing of these phases strongly depends on seasonal patterns of cumulative irradiation along latitudinal gradients. We demonstrated that maximal solar radiation is a suitable signal to determine the latitudinal time shift in the beginning of sensitive phases by a simple algorithm. A time shift amounting to 2.2 days per one degree of latitude was validated by a significant relation between latitude and timing of exceeding threshold temperatures defining the start of sensitive phases (Fig. 4). This result confirmed findings of Livingstone (2008) that solar radiation, the ultimate astronomical driving variable, is responsible for the most obvious spatially coherent fluctuations in lake surface temperature. Actually, our approach is verified for dimictic systems in Europe along a latitudinal gradient from 45 to 60°N. It needs to be confirmed whether our approach may be used within a wider latitudinal and longitudinal gradient, higher altitudinal ranges or other diverging boundary conditions.

Epilimnion temperatures in Saidenbach Reservoir during the four phenomenological phases are controlled by cumulative irradiation and air temperature, while the NAO influences epilimnetic temperature only temporarily (Table 3). This result confirms earlier findings that apart from winter and early spring, variability in water temperature is often not directly related to variability in the NAO (Gerten and Adrian 2000; Wagner and Benndorf 2007). A heat-budget model developed by Arhonditsis et al. (2004) indicated that global irradiation (short- and long-wave radiation) and changes in radiative heat exchange are main factors causing the interannual variability in thermal properties observed in this type of ecosystem. From the correlations with irradiation, we conclude that the four phases are indeed climate-sensitive as required and, compared to NAO capture climate signals beyond the winter--spring transition. This, we suppose is particularly important for studying the second aspect of sensitivity concerning the effect of sensitive phases, for example phenology, which for many pelagic organisms is related to the onset of stratification (Weyhenmeyer et al. 2008; Thackeray et al. 2010; Horn et al. 2011; see section *Apply CW*-*codes to predict warming trends and ecological responses*).

### Threshold temperatures separating cold from warm phases

Our approach enables a simple classification of warm and cold patterns during climate-sensitive phases, adding a useful tool to the suite of approaches that are currently used to analyse climatic impacts on dimictic aquatic systems. The threshold values represent averages of mean phase-specific water temperatures for each study site after accounting for a latitudinal time shift. Hence, they relied on natural climatic year-to-year variability during the last two decades, representing a period with a consistent warming trend (Weyhenmeyer et al. 2004; Bates et al. 2008). Temperature thresholds are virtually identical between the geographically distant lakes supporting findings of Livingstone et al. (2010) that surface water bodies can be viewed as local samples of a climatically driven continuum. Nevertheless, the coherence in epilimnion temperatures was surprising in view of the large distance separating the lakes (1,500 km) and also regarding the fact that the lakes differ in morphometry and altitude (Table 1). However, Livingstone and Padisak (2007) similarly found that fluctuations in epilimnetic temperatures mirrored fluctuations in the smoothed regional air temperature over distances of about 700 km.

Despite the general match in mean temperatures between lakes, there are differences in the degree of coherence between the four sensitive phases. Similarity in mean temperatures is lowest during inverse stratification in winter (phase 1) when fluctuations in surface temperatures are small and are strongly controlled by the occurrence of an ice cover (Weyhenmeyer et al. 2004; Arvola et al. 2010). In contrast to mountainous regions above about 2,000 m, where surface water temperature can be decoupled from ambient air temperature (Livingstone and Dokulil 2001), altitudes up to 500 m did obviously not affect the spatial coherence of epilimnion temperatures in our study. During spring overturn, mean epilimnion temperatures matched very well among lakes from 47 to 59°N (Fig. 5). During summer, however, geographic latitude may influence the maximum epilimnion temperatures as shown also by Fang and Stefan (1999). Temperatures observed at stations in the North Sea and in the more brackish Baltic Sea were higher or similar to lakes during winter and spring overturn. During the early thermal stratification and summer, however, marine sites tended to respond less sensitive to irradiation than the lakes resulting in lower surface water temperatures in North and Baltic Sea (this study; Sharples et al. 2006). In general, internal factors such as salinity, tidal currents, water from the Atlantic Ocean or river runoff as well as wind field variability may affect the strength of the link between climate forcing and the thermal properties in the marine systems (Elliott and Clarke 1991; Eilola and Stigebrandt 1998; Tian et al. 2011).

### Cross-system coherence in CW-codes among lakes at different latitudes and coastal marine systems

We found a high temporal and spatial coherence in CW-codes between the six dimictic lakes (within a latitudinal gradient from 47 to 59°N) even by using similar temperature thresholds to separate cold from warm sensitive phases. As all relationships between the reference lake and the other limnetic systems were highly significant (Table 5), we conclude that combining phase-specific threshold temperatures (discussed above) and long-term data of water temperature with a high temporal resolution results in a reliable estimate to cold-warm pattern for single phases, years and study sites. With respect to the temporal resolution of temperature data, we found that time-weighted means of epilimnetic temperatures calculated from daily measurement is best of course, but obviously, fortnightly temperature data supplemented by values determined shortly before and after the specific phase proved to be sufficient to classify phases as warm or cold. The high spatial coherence confirmed also that separating cold from warm patterns by normative values might be a very robust tool for screening of long-term data sets and for prediction. After pooling the data of all systems and years used here, we did no longer detect an impact of latitude, altitude, morphometry or salinity on the cold--warm pattern among the systems. This result provides evidence that our simple approach considers important large-scales drivers as postulated by Magnuson et al. (1990); Livingstone (2008) and Weyhenmeyer (2008).

If we compare coherence between the four sensitive phases, we found highest similarity (with 91 % of the observed inter-annual variance in warm--cold pattern between all lakes) during spring overturn. This implies that local effects on spring overturn are notably marginal compared to large-scale drivers supporting findings by Bluszcz et al. (2008) that the impact of net radiation is maximal in spring. As spring overturn occurred not until May in Lake Erken (Weyhenmeyer et al. 1999) but already in the beginning or middle of April in Saidenbach Reservoir (Rolinski et al. 2007), applying the latitudinal time shift is, however, an essential precondition to reach a high degree of coherence in CW-codes between distant lakes. Concerning the winter period, climate forcing supplies the basic structure of spatial coherence, but the local differences between lakes exceeded values observed during spring overturn. Variances in CW-pattern during winter depend strongly on the establishment and duration of an ice coverage as postulated also by Adrian et al. (1999) and Weyhenmeyer (2008). In Saidenbach Reservoir, there is a lot of variability between years ranging from no ice cover to an ice-off as late as Mid-April (Rolinski et al. 2007), which is in complete accordance with CW-patterns observed in Lake Scharmützelsee during winter (J. Rücker personal communication). A high degree of coherence in the timing of ice coverage was also observed among 196 Swedish lakes spanning a large latitudinal gradient (from 55°N to 68°N), which was significantly related to the winter NAO and to the regional atmospheric circulations (Weyhenmeyer et al. 2004). In contrast to all other lakes of our study, Lake Erken (representing the lake with the highest latitude) is regularly covered with ice from late December to late April (Weyhenmeyer et al. 1999). Thus, at the actual stage of climate warming, winter is never classified as warm in Lake Erken resulting in an absolute similarity to Saidenbach Reservoir for cold-winter years but in no accordance during warm-winter years (Fig. 6a). Lake Greifensee (representing the lake with the lowest latitude) was ice covered in about half of the winters of the last century (Franssen and Scherrer 2008). Nevertheless, in Lake Greifensee, warm-winter years matched by 100 %, and cold winter years still by 75 % to our reference lake.

The patterns of warm conditions during early thermal stratification matched in 85 % of years averaged over all lakes. According to studies of George et al. (2000) and of Livingstone and Dokulil (2001), regional coherence in meteorological driving forces (e.g., wind speed) contributes to the coherence between lakes by determining the timing and stability of thermal stratification. Deep lakes tend to exhibit a more persistent physical response to climatic forcing than shallow lakes (George et al. 2000; Gerten and Adrian 2001). Thus, epilimnion temperature in Bautzen Reservoir can transitionally be decoupled from ambient irradiation at temporary wind mixing events (Wagner and Benndorf 2007), which may explain the relatively low coherence to the deep Saidenbach Reservoir (Fig. 6a) during summer. In general, however, similarity in the CW-codes among the lakes is not significantly affected by mean depth as long as the analysis is limited to dimictic water bodies. Despite the relatively high latitude of Lake Erken, threshold temperatures for warm summer years (>20 °C derived from all lakes) were exceeded at least in 5 years of the long-term data set. In Lake Greifensee, similarity also decreased during summer as maximum surface water temperatures increased due to the low geographic latitude (Fang and Stefan 1999). In general, the lower coherence observed during summer indicates that this phase is less climate-sensitive than winter, spring overturn or early thermal stratification. This result supports findings of Livingstone and Dokulil (2001) that during summer variability in water temperatures is related to climate forcing on local rather than global scales.

In spite of very different hydrographic characteristics between the dimictic lakes and the Baltic Sea, relationships in CW-codes between each of the five stations in the Baltic Sea and the reference lake were significant even when applying threshold temperatures derived from lakes also for the marine system. CW-codes determined in the Baltic Sea were congruent with cold--warm patterns estimated in the reference lake with 85 % during winter and spring overturn implying that the intensity of winter cooling controls the spring overturn in the southern part of the Baltic Sea (Alheit et al. 2005). Eilola and Stigebrandt (1998) showed that the start of the spring stratification is related to the run-off of warm freshwater in the central area of the Baltic Sea. A strong seasonal thermocline develops over large parts of the Baltic Sea simultaneously to the lake sites during May and June (Schiewer 2008). A similarity to lakes of roughly 100 % for Kiel Bight during the period from winter through early thermal stratification indicates comparable thermal properties for both systems. In contrast to the deep reference lake, thermal stratification was temporarily interrupted in the Kiel Bight (e.g., 2005 or 2010, C. Clemmensen personal communication) reducing the coherence during summer. As shown by Hinrichsen et al. (2007), during summer, marine sites tend to respond generally less sensitive to irradiation than dimictic lakes due to the higher thickness of the mixed layer resulting in a slower increase in surface water temperatures. Surprisingly, using specific temperature thresholds for the Baltic Sea for determining CW-codes would result only in a slight increase in the coherence to lakes for Kiel Bight and Oder Bank, while the predictability of the warm--cold pattern increased considerably for the Arkona Basin, Darss Sill and Fehmarn Belt (Table 6).

Contrary to findings related to the Baltic Sea, CW-codes determined in the North Sea generally deviated somewhat from the regional coherence as warm--cold patterns matched only by 47 to 72 % with the reference lake codes when using lake-specific temperature thresholds (Fig. 6b). Due to the high level of salinity, most areas of the North Sea are not ice covered but vertically well mixed during winter (with the exception of very cold winter periods, e.g., 1996). Therefore, no inverse stratification occurs, and mean water temperatures during winter and spring are higher compared to all other study sites (Fig. 5). The low similarity during phase 3 is most likely the result of multi-scale fluctuations in timing and magnitude of river runoff (e.g., Ems, Weser, Elbe) and water column stratification in the North Sea (Tian et al. 2011). The development of a thermal stratification is controlled not only by surface solar irradiance and wind stress (as observed in lakes), but also by the competing mixing driven by tidal currents, salinity and convective overturning (Eilola and Stigebrandt 1998; Sharples et al. 2006; Tian et al. 2011). Nevertheless, increasing air temperatures have driven also a gradual trend in the timing of stratification in the North-western North Sea (Sharples et al. 2006) as observed in lakes. By applying site-specific threshold temperatures for the North Sea (Table 4), the comparison with the warm--cold pattern determined in the reference lake showed a remarkably similarity (Table 6), with 82 % of the cases (Fig. 6c). This result provides evidence that large-scale meteorological drivers (such as solar radiation, air temperature and wind speed) have also a significant influence on the seasonal pattern in the temperature development of the North Sea as demonstrated earlier by Elliott and Clarke (1991) and Sharples et al. (2006).

### Apply CW-codes to predict warming trends and ecological responses

When applying the CW-concept for the analysis of long-term trends in the frequency of warm phases, the observed positive trend is in agreement with results of trend analyses by the nonparametric, rank-based Mann–Kendall test in Saidenbach Reservoir (L. Paul, unpublished data) and other lakes (Gerten and Adrian 2001; Arhonditsis et al. 2004; Arvola et al. 2010) demonstrating strongest warming during April and May. Compared to “traditional” temperature trend analyses, our approach provides a closer relation to patterns of warming during specific phenomenological phases (Fig. 3). Despite the lower slope of logistic equations describing the trend in the probability of occurrence of warm patterns for winter, spring overturn and summer stagnation (compared to early thermal stratification), the probability of the occurrence of warm phases will exceed 80 % for all phases in 2030. Besides the general warming trend during early thermal stratification, we found an increase in the frequency of years with two and three warm phases per year since 1975 and since 2008 even of years with four warm phases that never occurred before 2008 (Fig. 3a) indicating a clear response to recent climate warming on dimictic systems during specific phenomenological phases. Our study demonstrated clearly that warming is not a continuous process, neither with respect to the seasonal pattern nor to the long-term trends.

Our generalized approach enables not only cross-system analysis of cold--warm patterns but also allows analysing coherence of other abiotic variables or of ecological responses to recent climate warming. We suggest that combining the cold--warm patterns of climate-sensitive phases with statistical box-plot analysis of abiotic and biotic criteria provides a simple tool to test ecological sensitivity of these criteria to specific warming patterns. As an example, we analysed the Brunt--Väisälä frequency in July and found a significant effect of warming both during winter and summer stagnation (Fig. 7a). When the epilimnion temperature during summer stagnation is high, this directly increases the stability frequency (compare Arvola et al. 2010). Besides, we identified an indirect negative (time delayed) effect of elevated winter temperatures on the stability frequency of the upper water column in July due to stronger warming of the hypolimnion water during winter, which is often extended to spring and summer by a memory effect (Gerten and Adrian 2001; Dokulil et al. 2006). Phases 2 and 3 are considered to be less sensitive with respect to the stability frequency during summer. As shown by Blenckner et al. (2007), a higher stability frequency during summer or an extension of the stratified period may indirectly set the boundaries for the structure of future summer plankton communities.

The analysis of *Daphnia* biomass during summer in relation to climate-sensitive phases illustrates that especially phase 3 is also ecologically sensitive causing effects on ecological variables and processes. Warming during early thermal stratification rather than winter (Hülsmann et al. in press) or summer temperatures may control food-web-mediated effects on the population dynamics of the key plankton species during summer beyond those expected from direct effects (Benndorf et al. 2001). The temperature signals of the four sensitive phases are indeed transmitted into the physiology and phenology of *Daphnia*; in addition however, they undergo a time-delayed response of higher trophic levels (YOY-fish and invertebrate predators) changing trophic interactions. For mechanistic understanding of the food-web-mediated temperature effects, see detailed analysis given in Wagner et al. (in press) and Wagner and Benndorf (2007). Thereby, even a subtle, moderate warming could tilt the balance in predator–prey interactions and lead to far-reaching ecosystem-wide consequences (Emmerson et al. 2005). In addition to other approaches (Straile 2000; Stenseth and Mysterud 2002; Adrian et al. 2006), our phenomenological-driven categorical analysis allows to differentiate easily between instantaneous and time-delayed ecological responses to seasonal warming pattern. As our concept also takes latitudinal gradients in the timing of phenomenological phases into account, a regional coherence in the response at the biological level between ecosystems may be supposed as an indirect result of coherence in the primary physical response as postulated by Livingstone et al. (2010).

The knowledge of differences in ecological responses is essential to predict climate-driven changes in trophic cascades and ultimately in lake ecosystems under near-future warming scenarios. Further studies should focus on the comparison of ecological responses to warming patterns during sensitive phases among dimictic aquatic systems. Our approach may facilitate not only cross-system analysis of climate effects on water temperature patterns and on direct and indirect ecosystem response during sensitive phases, but provides also the basis for prediction of warming trends and defining climate scenarios for experimental or modelling studies in a more specific way than only increasing the long-term average temperature.
